# Loss of Function of the Retinoblastoma Gene Affects Gap Junctional Intercellular Communication and Cell Fate in Osteoblasts

**DOI:** 10.3390/biology13010039

**Published:** 2024-01-11

**Authors:** Elisha Pendleton, Anthony Ketner, Phil Ransick, Doug Ardekani, Thomas Bodenstine, Nalini Chandar

**Affiliations:** 1 Department of Biochemistry and Molecular Genetics, College of Graduate Studies, Midwestern University, Downers Grove, IL 60515, USA; ependl@midwestern.edu (E.P.); aketner77@midwestern.edu (A.K.); tboden@midwestern.edu (T.B.); 2 Chicago College of Osteopathic Medicine, Midwestern University, 555 31st Street, Downers Grove, IL 60515, USA; prasnick76@midwestern.edu (P.R.); dardekani54@midwestern.edu (D.A.)

**Keywords:** Retinoblastoma gene, osteoblast differentiation, gap junctional intercellular communication, connexin 43

## Abstract

**Simple Summary:**

Certain tumor suppressor genes, such as p53 and Retinoblastoma gene (RB1), that normally function to maintain order and prevent the occurrence of cancer in normal tissue are usually found to be mutated at a high rate in bone cancers (osteosarcomas). In this manuscript we have investigated some of *Rb1*’s functions by creating a deficiency in the expression of the Rb1 in bone cells to determine how this affects their ability to maintain bone-specific gene expression and function. We found that loss of Rb1 function in cells predisposed them to express features of both adipocytes (fat cells) and osteoblasts (bone cells). Normally, when these osteoblasts are induced to grow and function specifically as bone tissue, they significantly increase their communication with their neighboring cells. When the Rb1 gene function is reduced, there is a loss of this communication. The membrane protein essential to mediate this communication, connexin 43 (Cx43), is also reduced. One of the reasons why loss of Rb1 function might cause a change in the behavior of osteoblasts, relates to the increased expression of a master regulator of adipocyte formation—a protein called PPAR-gamma. We found the expression of PPAR-gamma to be increased by about 7–9-fold in Rb1-deficient cells. As this master regulator of adipocyte differentiation could potentially induce the changes seen, we reduced PPAR-gamma levels in control osteoblasts and Rb1-deficient cells to determine if the adipocyte features could be reversed. We found that normal osteoblasts and Rb1-deficient osteoblasts were able to revert to a predominantly osteoblast-specific expression when PPAR-gamma was reduced. However, the changes to communication and Cx43 expression were not reversed in Rb1-deficient cells. This suggests that either normal Rb1 function and/or other events in the process of osteoblast differentiation regulate appropriate cellular communication, and not PPAR-gamma in normal bone cells.

**Abstract:**

Loss of function of the Retinoblastoma gene (RB1) due to mutations is commonly seen in human osteosarcomas. One of the Rb1 gene functions is to facilitate cell fate from mesenchymal stem cells to osteoblasts and prevent adipocyte differentiations. In this study, we demonstrate that a stable reduction of Rb1 expression (RbKD) in murine osteoblasts causes them to express higher levels of PPAR-ɣ and other adipocyte-specific transcription factors while retaining high expression of osteoblast-specific transcription factors, Runx2/Cbfa1 and SP7/Osterix. Inhibition of gap junctional intercellular communication (GJIC) in osteoblasts is another mechanism that causes osteoblasts to transdifferentiate to adipocytes. We found that preosteoblasts exposed to osteoblast differentiating media (DP media) increased GJIC. RbKD cells showed reduced GJIC along with a reduction in expression of Cx43, the protein that mediates GJIC. Other membrane associated adhesion protein Cadherin 11 (Cad11) was also decreased. Since PPAR-ɣ is increased with Rb1 loss, we wondered if the reduction of this transcription factor would reverse the changes observed. Reduction of PPAR-ɣ in control osteoblasts slightly increased bone-specific expression and reduced adipocytic expression as expected along with an increase in Cad11 and Cx43 expression. GJIC remained high and was unaffected by a reduction in PPAR-ɣ in control cells. Knockdown of PPAR-ɣ in RbKD cells reduced adipocyte gene expression, while osteoblast-specific expression showed improvement. Cx43, Cad11 and GJIC remained unaffected by PPAR-ɣ reduction. Our observations suggest that increased PPAR-ɣ that happens with Rb1 loss only affects osteoblast-adipocyte-specific gene expression but does not completely reverse Cx43 gene expression or GJIC. Therefore, these effects may represent independent events triggered by Rb1loss and/or the differentiation process.

## 1. Introduction

Osteosarcomas are characterized by mutations and inactivation of p53 and retinoblastoma (RB1) tumor suppressor genes [1,2]. In our studies with murine osteosarcoma, we have been able to show a role for p53 in the regulation of osteoblast differentiation through expression of bone-specific genes [2,3,4]. Wild type p53 gene function does not appear to be necessary for development, but mice heterozygous for p53 are more prone to osteosarcomas [5]. Osteoblast-specific gene expression and differentiation are also affected by a reduction in p53 dosage, demonstrating that p53 regulates bone remodeling by targeting bone-specific genes, and this function is affected by a deficiency of p53 [6]. While p53 function is important in postnatal bone, Rb1 function has been shown to be developmentally important, as mice with germline Rb1 knockouts are embryonic lethal [7].

The Rb1 gene belongs to the pocket protein family with sites for functional binding of other proteins [8]. Patients with familial retinoblastoma are heterozygous for a Rb1 mutation with subsequent loss of the remaining allele producing the pediatric eye tumor retinoblastoma. These patients often go on to develop secondary tumors in their bones (osteosarcomas) [9], highlighting the importance of Rb1 dosage and function in bone growth and differentiation. Other than Rb1’s established role in cell cycle control, new roles have been ascribed for this gene in the regulation of chromatin, apoptosis, differentiation [10]. In the context of tissue differentiation, Rb1 is important for cell fate determination, especially for osteoblast fate from mesenchymal stem cells [11]. Another function that has been attributed to Rb1 is in cell adhesion [12,13]. It has also been known that Rb1 binds and potentiates Runx2 (Cbfa1), a bone-specific transcription factor [14] while inhibiting PPAR-ɣ an adipocyte-specific transcription factor [11].

Targeted deletion of p53 and Rb1 in uncommitted mesenchymal cells leads to osteosarcomas with expression of multi-lineage differentiation markers [11]. Tumor spectrum also appears to differ depending on whether there is only p53 loss or both p53 and Rb1 loss, with the latter producing an expanded spectrum of tumors (osteosarcomas, hibernomas, sarcomas, etc.) [11]. This would suggest that a loss of Rb1 predisposes mesenchymal stem cells away from the osteoblastic fate or causes their dedifferentiation.

Another mechanism that causes osteoblasts and other mesenchymal cell types to default to an adipocytic phenotype is when gap junctional intercellular communication (GJIC) is inhibited [15,16]. These studies have mainly employed chemical inhibitors of gap junctional communication even though connexins are gap junctional proteins that allow intercellular communication and the movement of ions and signaling proteins between cells [17]. One of the main gap junctional proteins in osteoblasts is connexin 43 (Cx43) aka gap junction protein alpha 1 (GJA1) which increases in abundance as cells undergo differentiation. Cx43 affects the expression of bone-specific transcription factors such as Runx2, osterix/Sp7 and bone-specific proteins such as Type 1 collagen and osteocalcin [14,18,19]. Mutations in Cx43 gene are associated with oculodentodigital dysplasia and other skeletal manifestations in humans [20].

The importance of Cx43 in maintaining osteoblast function and differentiation is well established [21,22] and the role of GJIC in maintaining tissue-specific differentiation has also been confirmed by other independent studies without directly implicating connexins. GJIC can be measured in vitro using techniques that rely on preloading cells with fluorescent probes and dyes, which can migrate from loaded cells to adjacent cells [23,24,25].

While recent studies have suggested a role for pRb in cell adhesion and osteoblast cell fate [8,11,12], it is unclear if osteoblast specific GJIC is maintained with Rb loss. No studies have investigated if Rb1 plays a role in maintaining GJIC function. In this study, we aimed to explore the possibility that losses of Rb1 and GJIC function may be linked.

## 2. Materials and Methods

### 2.1. Cell Culture and Conditions

The MC3T3-E1 subclone 14 mouse osteoblast-like cells originally isolated from mouse calvaria, were obtained from ATCC (ATCC-CRL-2593) and used for experiments. Cells were grown in 10% FBS (Fetal Bovine Serum) and αMEM media.

### 2.2. Cell Lines and Treatment

MC3T3-E1 cells were used to create Rb1-deficient line using shRNA technology and have been described earlier [26]. This clone was generated using a mixture of two validated shRNAs—a kind gift of Dr. Alexandrow [27]. A stable clone of cells containing less than 40% of wild-type Rb1 expression was used for these studies (RbKD). Other clones using either shRNA also produced a similar reduction (40–60%) and were used for confirmation of our results. The control MC3T3-E1 line contained a stably expressing scrambled shRNA and will be referred to as control cell line. For transient knockdown of Rb1 we performed the transfection followed by exposure to AP or DP media 24 h later (day 0).

The PPAR-ɣ shRNA (Santa Cruz Biotechnology, Dallas, TX, USA) was used to create transient and stable knockdown of PPAR-ɣ within the MC3T3-E1 cells. Effectene (Qiagen, Germantown, MD, USA)) was used for both transient and stable transfections according to the manufacturer’s protocol. Additionally, for obtaining stable clones, cells were selected post-transfection using puromycin. Single cell clones were expanded, passaged, and characterized before use. For generation of cells double knockdowns, we transfected RBKD cells with the PPAR-ɣ shRNA as described above. For selection of clones, we carried out serial dilution after puromycin selection to obtain single cell clones. These clones were expanded and tested for the level of PPAR-ɣ loss. Cells with at least 50% knockdown were selected for our studies. Care was taken to choose clones with comparable knockdown of PPAR-ɣ from both cell types of control and RbKD.

C2C12 OSX Tet-off cell line was a gift from Dr. K. Sinha [28]. 80 ng/mL of tetracycline (Sigma-Aldrich, St. Louis, MO, USA) was added to the growth media to maintain inhibition of osterix expression.

For induction of a specific differentiation pathway, the following media were used and are known to hasten the expression of the respective cell type [26].

Adipocyte differentiation promoting media (AP media): The AP media used consisted of 2% FBS (Fetal Bovine Serum) and αMEM containing 0.5 mM 3-isobutyl-1-methylxanthine (Sigma-Aldrich, St. Louis, MO, USA), 1 μM dexamethasone (Sigma-Aldrich, St. Louis, MO, USA), 10 μg/mL Insulin (Sigma-Aldrich, St. Louis, MO, USA). The AP media were changed every 48 h after the initial treatment, and the cells were maintained in an incubator at 37 °C and 5% carbon dioxide.

Osteogenic differentiation promoting media (DP media): For studies exploring differentiation, cells were treated with the differentiating medium (DP media) consisting of basal Alpha -MEM medium containing 50 μg/mL ascorbic acid (Sigma-Aldrich, St. Louis, MO, USA) and 100 mM biglycerol phosphate (Sigma-Aldrich, St. Louis, MO, USA).

For the chemical inhibition of communication, cells were treated for four days with either 100 μM 18-a-glycyrrhetinic acid, (AGRA) (inhibitor) (Sigma-Aldrich, St. Louis, MO, USA), 100 μM glycyrrhetinic acid, (GA) (non-inhibitor analog) (Sigma-Aldrich, St. Louis, MO, USA) or DMSO (Sigma-Aldrich, St. Louis, MO, USA) in regular growth media.

### 2.3. Quantitative Realtime PCR

RNA was isolated using TRI reagent (Sigma-Aldrich, St. Louis, MO, USA). A Two-step RT-PCR (reverse transcription polymerase chain reaction) was performed using Bright Green qPCR master Mix (Applied Biological Materials, Richmond, BC, Canada) and One Script Plus Reverse Transcriptase with Step One Plus Real Time Machine (Applied Biological Materials, Richmond, BC, Canada) RNA samples were converted into cDNA, using a high-capacity cDNA RT kit. Analysis of the results was performed with the 7300 RT-PCR System RQ Software version 1.4 (Thermo Fisher Scientific, Waltham, MA, USA). Efficiency of the control and treatment samples were checked using the slope of the graph of ΔCT values versus log of the total RNA sample. If the calculated slope was less than 0.1, then comparative CT method was used to compare the gene expression between control and treatment cells. These were normalized according to a housekeeping gene, β-actin, serving as an internal control (Table 1).

### 2.4. Western Blot Analysis

Cells were lysed with buffer, containing 50 mM DTT, 2% SDS, 10% glycerol, 65 mM Tris–HCl pH 6.8, with protease inhibitor cocktail (cOmplete ULTRA Tablets Mini Roche protease inhibitor). The lysate was collected and sonicated with a Bioruptor 300 (Diagenode, NJ, USA). Protein concentrations were measured, and a standard curve was generated using a Pierce BCA Protein Assay Kit (Thermo Fisher Scientific, Waltham, MA, USA) SuperSignal^®^ West Pico Chemiluminescent Substrate (Thermo Fisher Scientific, Waltham, MA, USA) was used to visualize the membrane using BioRad ChemiDoc MP Imaging System (BIORAD, Hercules, CA, USA). Mouse Gapdh (Proteintech, Rosemount, IL, USA), Rabbit Connexin43 (Proteintech, Rosemount, IL, USA), Rabbit Cadherin 11 (Thermo Fisher Scientific, Waltham, MA, USA), Rabbit PPARG (Cell Signaling Technology^®^, Danvers, MA, USA).

### 2.5. Immunofluorescence

Intracellular localization of pRb1 and Cx43 was imaged using immunofluorescence and confocal microscopy. Cells were seeded on coverslips and added to wells of a 24-well plate. The cells were exposed to DP media for 0, 1, 2, 3, and 4 days and allowed to grow to confluency and fixed. Primary antibody (Rabbit Cx43 Ab (1:500): Cell Signaling Technology^®^, Danvers, MA, USA; beta-Actin mouse mAb (1:2000): Cell Signaling Technology^®^, Danvers, MA, USA) was added to the wells in 2% BSA/PBS and incubated overnight at 4 °C while shaken. Secondary antibody (Alexa Fluor™ 488 goat anti-rabbit IgG -1:500): (Thermo Fisher Scientific, Waltham, MA, USA); Alexa Fluor™ 594 goat anti-mouse IgG (1:5000): (Thermo Fisher Scientific, Waltham, MA, USA) was added to the wells in 2% BSA/PBS and incubated at 4 °C while shaken for 3 h. Coverslips were mounted on glass slides with Fluoroshield™ with DAPI (Sigma-Aldrich, St. Louis, MO, USA). Slides were analyzed with confocal microscopy using a Nikon A1R and Nikon Eclipse Ti inverted microscope and (Nikon Instruments Inc., Melville, NY, USA).

### 2.6. Dye Transfer GJIC Assay

For GJIC analysis, cells were analyzed and quantified by flow cytometry [24,25] Briefly, donor cells were labeled with 0.5 µM of the lipophilic permanent dye Cell Tracker Deep Red (Thermo Fisher Scientific, Waltham, MA, USA) and 0.5 µM calcein-AM (Thermo Fisher Scientific, Waltham, MA, USA) and cultured with non-labeled cells. Spread of calcein from donor cells (Deep Red/calcein positive) to acceptor cells (calcein only) indicated GJIC activity as demonstrated with the highly gap junction coupled in MC3T3 E1 and RbKD lines. Quantification of GJIC was performed using a Beckman Coulter CytoFLEX Flow Cytometer (Beckman Coulter, Brea, CA, USA) flow cytometer with Kaluza Analysis software 2.1. Coupling efficiency was represented as the level of GJIC activity and calculated as acceptor cells/donor cells and normalized to control.

### 2.7. Statistical Analyses

All experiments were conducted three times and data were presented as mean ± standard deviation (SD) of triplicate samples. Statistical analyses were done using GraphPad Prism 9.00 (GraphPad Software, Inc., San Diego, CA, USA). Comparisons between groups were made using the student’s *t*-test or one-way multivariate analysis of variance (ANOVA) followed by Tukey’s multiple comparison test. *p* < 0.05 was considered to indicate a statistically significant difference.

## 3. Results

### 3.1. Reduction in pRb Expression in Osteoblasts Produces Changes to the Cell’s Phenotype

**Figure 1:**
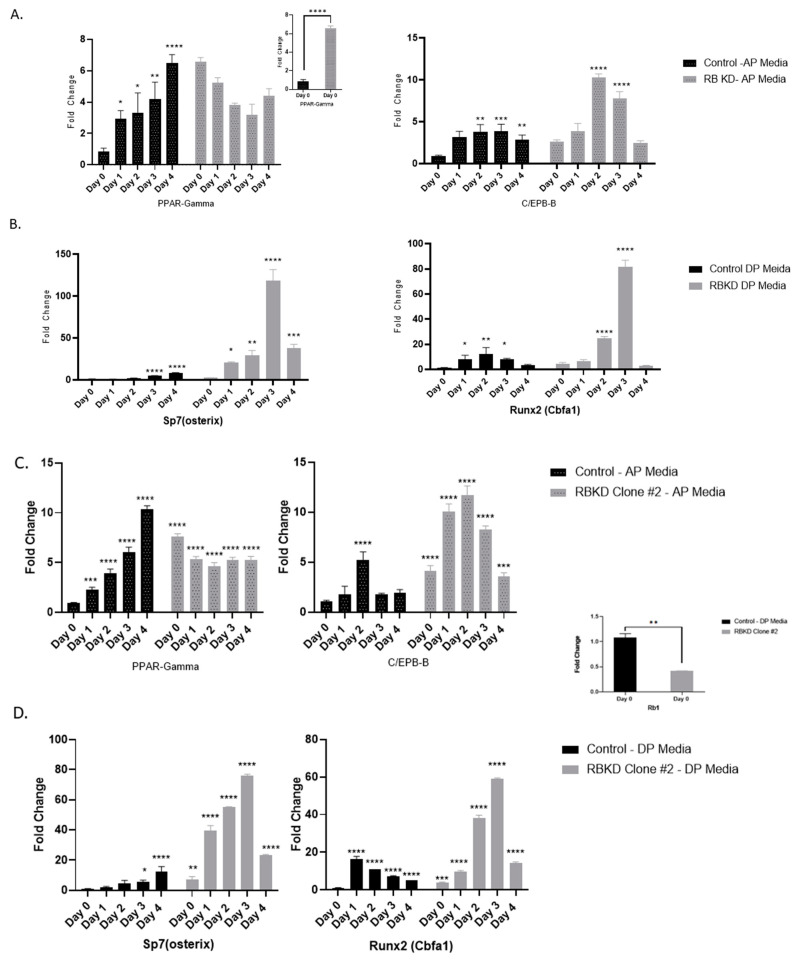

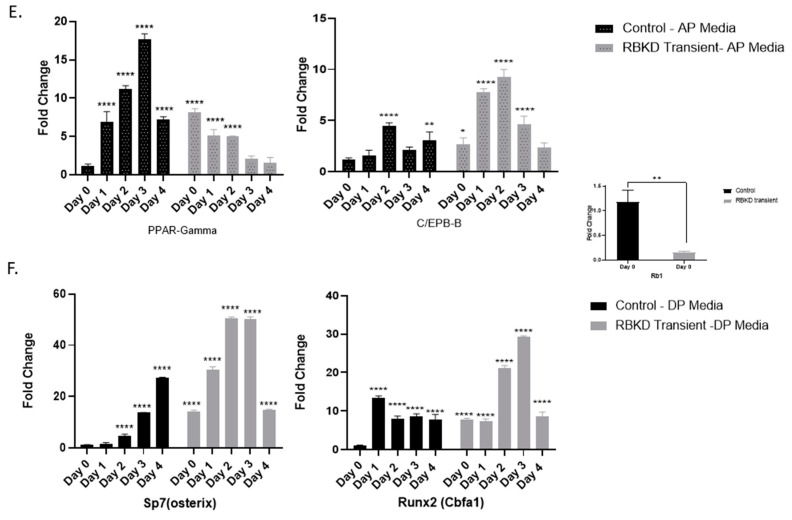
Comparison of adipocyte and osteogenic transcription factor expression following exposure to AP (**A**) or DP (**B**) media. (**C**–**F**) represent similar experiments done with a clone using shRNA2. These cells showed a similar knockdown as our main RBKD cell line of about 60% (**C**,**D**) inset). (**E**,**F**) were generated using transient transfection with shRNA1. These experiments yielded an Rb1 knockdown of 80% (inset). All experiments represent an average of three separate measurements. **** *p* < 0.0001, *** *p* < 0.001, ** *p* < 0.01 * *p* < 0.05 compared with day 0.

For our experiments, we have used determined preosteoblasts MC3T3E1 and the same cells with a stable Rb1 reduction (60% reduction of endogenous Rb1) obtained using a mixture of shRNA1 and shRNA2. These cells have been described in previous studies [26] and other details are available under methods. We wanted to determine if specific transcription factors that control adipocyte versus osteoblast differentiation are modified by the loss of Rb1 expression. Cbfa1 (Runx2) and SP7/Osterix are two transcription factors that are necessary to induce osteoblast differentiation and have been shown to regulate the transcription of downstream osteoblast differentiation genes [29,30]. PPAR-ɣ is the master transcription factor and it works in concert with different C/EBPs in the regulation of adipocytic differentiation pathway [31]. Earlier work has shown targeted loss of Rb1 to produce these effects [11,32] and we wanted to confirm if this can be extended to osteoblasts with reduced Rb1 function. Control and Rb1-deficient cells (RbKD) were exposed to an adipogenic (AP), or osteogenic differentiation media (DP), as described in the methods section, for different lengths of time and the cell type-specific transcription factor expression was monitored. As shown in Figure 1A, control cells exposed to AP media showed changes consistent with differentiation towards the adipocytic lineage, as evidenced by an increase in expression of the master regulator for adipocytic differentiation, PPAR-ɣ and C/EBP-beta [33]. The ability of determined osteoblast cells to transdifferentiate is known [34], and comparable results have been obtained in the past by us with these and other adipocytic markers [26]. pRb-deficient cells showed a robust increase in these markers when compared to the control osteoblasts. It was interesting to note that in RbKD cells, PPAR-ɣ levels were 7-fold higher than controls without treatment (day 0 levels) (inset), showing that loss of Rb1 expression was sufficient to activate adipocytic differentiation while deranging the normal osteoblast differentiation process. Similar increases (3-fold) were seen for CEBP-beta when compared to the control following exposure to ADIP media (Figure 1A). We have also previously shown that RbKD cells show an increase in the accumulation of fat, as measured by oil red O staining [26].

When similar experiments were done using osteogenic DP media, which hastens the expression of bone-specific gene expression, deposition of extracellular matrix and mineralization [3], we noted the expected changes that follow differentiation in control osteoblasts (increase in transcription factors Runx2 and osterix/Sp7) (Figure 1B). As in the case of AP media, RbKD cells showed dramatic elevation of osteogenic transcription factors when compared to the control. In order to confirm the specificity of our knockdown, we performed additional experiments using individual shRNAs. In one experiment, we used a stable clone created with just shRNA 2 (Figure 1C,D), and in another, we used a transient knockdown of Rb1 using shRNA1 followed by AP or DP treatment (Figure 1E,F). In the case of shRNA2 knockdown resulted in about 40% of pRb expression remaining in clone tested, while transient knockdown with shRNA1 produced about 80% knockdown of pRb. In both cases, the elevation of PPAR-ɣ with Rb1 loss was maintained (7.5-fold with shRNA1 and 8.2-fold with transient knockdown of Rb1 (RbKD Day 0). A similar trend in the other bone and adipocyte markers were also maintained, suggesting that our different knockdown strategies yielded specific and similar results in osteoblast gene expression. These results suggested that a reduction in Rb1 function caused these cells to either acquire an ability to be bipotential or that these determined osteoblasts were becoming less differentiated [11]. It is also likely that the loss of cell cycle regulation with Rb1 loss played a role in increasing cell density and the resultant increase in gene expression that we and others have observed [26,32].

### 3.2. Gap Junctional Intercellular Communication Increases with Osteoblast Differentiation

GJIC assay was performed as described in the methods section. In this assay, the cell population under study is divided into two fractions—one fraction of double labelled cells is mixed with nonlabelled cells. The two fractions are mixed and calcein dye transfers to the nonlabelled cells through gap junctions. The assessment of uptake is made with imaging and quantitated using flow cytometry [23].

**Figure 2:**
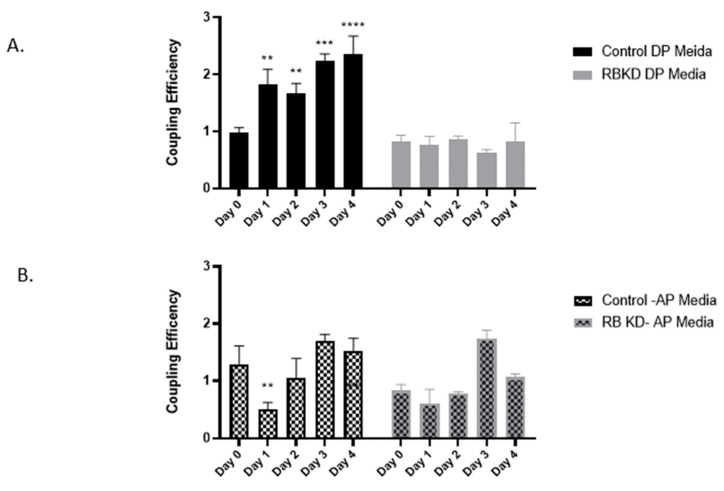
Quantitation of gap junctional intercellular communication (GJIC) in control and RbKD cells exposed to DP media (**A**) versus AP media (**B**). Donor cells were labeled with Calcein AM and CellTracker Deep Red and cocultured as described under methods. The spread of Calcein to nonlabelled cells was used as a measure of GJIC and evaluated by flow cytometry and quantitated. Values are represented as coupling efficiency changes when compared to 0-day control and are mean and standard deviation of three separate experiments. **** *p* < 0.0001, *** *p* < 0.001, ** *p* < 0.01 compared with day 0.

To demonstrate how GJIC changes with differentiation and the effect of reduction of Rb1 expression, we performed the dye transfer assay on MC3T3 E1 control cells induced to differentiate towards osteoblastic or adipocytic pathways. Cells were treated with DP media for the different time intervals and exposed to the dyes for the measurement of GJIC. Cells were then subjected to flow cytometry to assess the degree of coupling. In control osteoblasts, there was a 2–3-fold significant increase in gap junctional intercellular communication (GJIC) with increasing exposure to DP media (Figure 2A). A similar upregulation of GJIC during osteoblast differentiation was seen with our other shRNA clones.

This increase in communication has previously been established in several studies [35,36,37]. When RbKD cells were subjected to the dye coupling assay after a similar exposure to DP media, these cells showed negligible communication over time (right panel) suggesting that a reduction in pRb1 may have contributed to the reduction in GJIC. When the same cells were exposed to AP media, GJIC did not show a significant change during the differentiation process in control cells (Figure 2B). Cells with Rb1 deficiency exhibited a similar profile with no specific differences between them and control.

While GJIC is an important feature of osteoblast differentiation regulating not only gene expression but also the remodeling process [20,38,39,40], adipocyte differentiation is usually accompanied by a decrease in communication [41,42,43]. Some of these studies also show a corresponding decrease in the major gap junctional protein connexin 43 (Cx43) during this process [42,43].

To confirm the increase in GJIC as well as Cx43 expression during differentiation of osteoblast from precursor stem cells, we used a previously established model of osteogenesis, which uses C2C12, a bipotential cell line, where induced expression of osterix/Sp7 increases differentiation of myoblasts to osteoblasts [44]. Using this Tet off system, we allowed the induction of osterix/Sp7 which in turn activates the expression of bone-specific genes. Expression of bone-specific differentiation associated gene osteocalcin is shown as an example after three days in the absence of tetracycline (see inset in figure).

**Figure 3:**
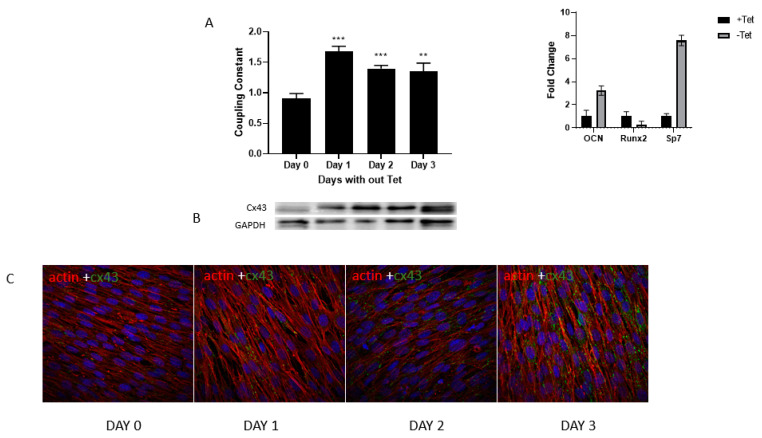
A stable C2C12 mesenchymal cell line in which overexpression of Sp7/OSX was induced by using the Tet-off system was employed. (**A**) These cells were exposed to media without Tet for the days mentioned and the dye transfer assay was performed. Values are represented as coupling efficiency changes when compared to 0-day control and are mean and standard deviation of three separate experiments. *** *p* < 0.001, ** *p* < 0.01 compared with day 0. An increase in bone-specific gene expression is indicated in the inset when measured on Day 1. (**B**) Changes to gene expression as measured by Western blotting and cellular distribution by immunofluorescence (**C**) using Cx43 antibody are shown for the same time intervals. DAPI was used to stain the nucleus. Magnification 60×.

Changes of GJIC during differentiation were monitored and quantitated using flow cytometry. There was a steady increase in GJIC over time, as evidenced by dye coupling when tetracycline is removed from the media for 1–3 days (Figure 3A). When Cx43 expression was determined in these cells, it was clear that an increase in GJIC was accompanied by an increase in Cx43 expression both in terms of the amount and in terms of localization to the membranes (Figure 3B,C). This suggests that this process is active at the level of determined preosteoblasts and at the progenitor level.

### 3.3. Inhibition of Gap Junctional Communication Increases Adipocytic Gene Expression in Osteoblasts

**Figure 4:**
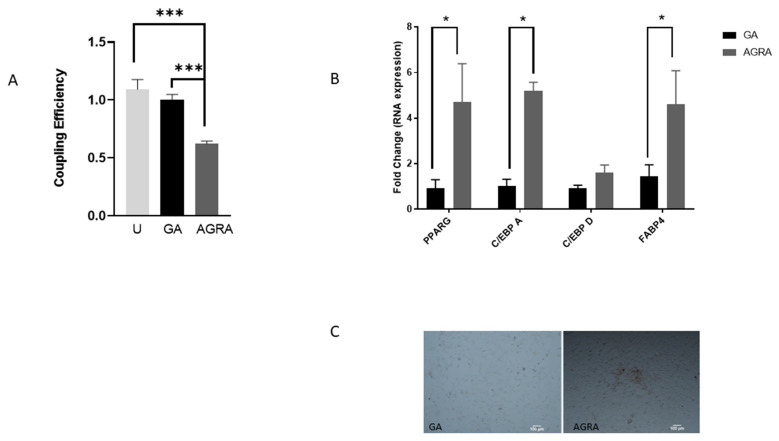
(**A**) Pre-osteoblast cells MC3T3-E1 were exposed to AGRA for 4 days as described in the methods section. The dye transfer assay to assess GJIC was performed. Cells received no treatment (U), a control non-inhibitor GA, or the GJIC inhibitor, AGRA. (**B**) Adipocyte-specific gene expression during this time was assessed using Realtime PCR. Values are represented as fold changes when compared to untreated control (**A**) or non-inhibitor GA (**B**) and are mean and standard deviation of three separate experiments. *** *p* < 0.001, * *p* < 0.05. (**C**) Oil red O staining to demonstrate lipid accumulation in cells after 4 days of GJIC inhibition.

Inhibition of gap junctional communication with the chemical 18-alpha glycerrhetinic acid (AGRA) has been previously shown to convert myocytes and osteoblasts to adipocytes [15,16]. We wanted to confirm this for our preosteoblast cell line MC3T3E1. We therefore exposed these cells to 18-alpha glycerrhetinic acid (AGRA) (inhibitor), glycerrhetinic acid (GA) (control, non-inhibitor) or no treatment (NT) for four days. After four days of incubation, cells were subjected to flow cytometry analysis to confirm inhibition of gap junctional communication. Results were compared to cells receiving no treatment (NT) or treatment with the non-inhibitor (GA). AGRA-treated cells showed a 40% drop in communication between cells when compared to cells treated with GA (Figure 4A). Additional samples treated similarly for four days with the inhibitors were used for RNA analysis. As shown in Figure 4B, the adipocytic transcription factors that are established to increase during adipogenesis show a significant increase in expression. The increase in PPAR-ɣ and CEBP-alpha occurs as a second wave and is seen here to be elevated 5–6-fold over controls. CEBP delta constitutes the first wave and has likely occurred at a point earlier than our 4-day AGRA treatment. FAB4 is an adipocyte marker that is expected to show increased transcription during differentiation. Figure 4C shows the accumulation of cytoplasmic lipid droplets in AGRA-treated cells. Our results show a shift to a more adipocytic phenotype has occurred due to inhibition of communication.

Our work here supports the observations that have been previously made regarding inhibition of communication and the ability of osteoblasts cells to express adipocyte-specific gene expression, as well as the fact that Cx43 is the predominant gap junctional protein that increases with osteoblast differentiation and aids in the communication process. However, while Rb1 loss predisposes cells towards the adipocytic lineage, little is known if Rb1 loss affects connexin 43 expression. A loss of Cx43 expression with Rb1 loss might suggest a role for Rb1 in gap junctional intercellular communication in osteoblasts. We therefore attempted to study gene expression during osteogenic versus adipocytic differentiation to determine if a role for Rb1 is likely.

### 3.4. Increase in GJA1 Gene (Cx43) Expression during Differentiation Is Not Seen in RbKD Cells

**Figure 5:**
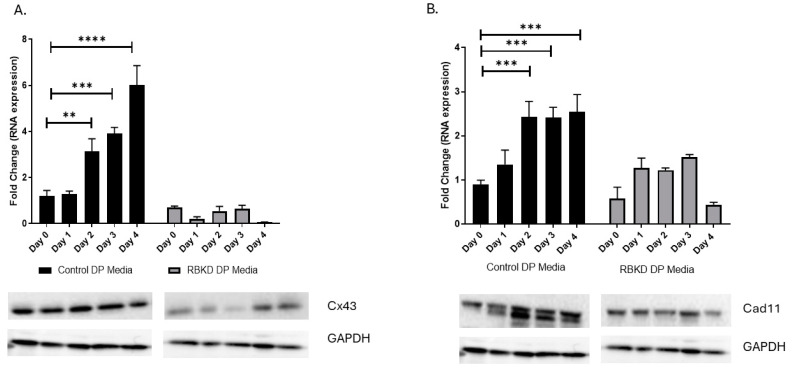

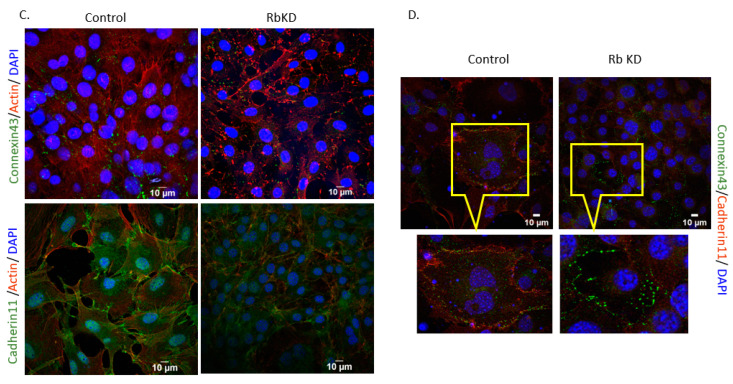
(**A**) Gene expression changes during osteoblast differentiation. Expression levels of Cx43 (**A**) and Cad11 (**B**) were evaluated after each time point by Western blot analysis. A representative blot and quantitation of three separate measurements are indicated. **** *p* < 0.0001, *** *p* < 0.001, ** *p* < 0.01, compared with day 0. (**C**) Immunofluorescence analysis of Cx43 and Cad11 expression in control and RbKD cells. DAPI blue, actin red, Cx43 green (top panel), Cad11 green (bottom panel). (**D**) Co-staining of Cx43 (green) and Cad 11 (red) demonstrates loss of association of the two proteins in RbKD cells (region magnified—inset).

Cx43/GJA-1 gene expression in DP media increased during the different time intervals ranged from 1–6-fold in control cells during the treatment period tested (Figure 5A). In the case of RbKD cells, at 0-time, the Cx43 expression was about 30% below control levels and did not increase significantly with time. Western blot analysis reflected changes that were expected based on the PCR (Polymerase Chain Reaction) data.

At the protein level, we visualized a 43 Kd band in both control and Rb KD lines. While there was an increase with differentiation in control cells, the variation seen in RbKD cells was minimal with time (Figure 5A). A quantitation of the changes noted after analyzing multiple Western blots confirmed the lack of changes in Cx43. These studies show a loss of Cx43 expression to be associated with a decrease in communication seen using the dye transfer assay.

We noticed changes in other membrane-associated proteins, such as zona occludens (ZO-1) (unpublished observations). The only evidence linking Rb1 to membrane-specific proteins in osteoblasts comes from work described by Sosa-Garcia et al. [12]. These investigators show deficiencies in the adhesive properties of osteoblasts lacking Rb1 [12]. As cadherin11 (Cad11) (aka OB-Cadherin, as it is abundant in osteoblasts) showed a decrease in their studies, we determined if the loss of Cx34 was also accompanied by the loss of Cad11. As shown in Figure 5B, there was a reduction in expression in RbKD cells without treatment and a more stunted increase in expression during differentiation when compared to the control.

We also determined the subcellular localization by conducting immunofluorescence staining. As shown in Figure 5C, we co-stained both proteins with an actin antibody. Cx43 expression was present on cell borders in control and RBKD cells and the amount varied as expected from our Western blot data. RbKD cells showed less staining at 0 time (shown) when compared to the control and this did not change dramatically with time as in the control differentiating cells (not shown). The same results were evident with Cad11 (lower panel). In both the cases, actin levels appeared to also be lower in RBKD cells. As the actin filaments and the actin cytoskeleton are important components in the processes of assembly and stabilization of gap junctions [45], their paucity in Rb1-deficient cells may also play a role in the proper assembly of connexins to form channels for GJIC or cadherins to form adherent junctions.

To determine how the reduced expression of the two proteins together might affect cell—cell interaction, we attempted to co-stain Cad11 with Cx43. Cadherins have been thought to facilitate the assembly of connexins (Cxs) into gap junctions by enhancing cell–cell contact. Gap junctions co-localize with these adherent junctions where cadherins associate with other proteins, such as β-catenin. As shown in Figure 5D, the colocalization of Cx43 with these proteins was easily visualized in control cells at the cell borders, as evidenced by a merging of the two colors used in staining. There was a close association of Cad11 positivity (red) with Cx43 (green) (left). In Rb KD cells, the reduction in Cad11 while present (Figure 5C, lower right) appears to have also affected the association with Cx43 (Figure 5D).

### 3.5. Reduction in PPAR-ɣ Levels Does Not Completely Reverse the Phenotype Produced with Rb2 Loss

**Figure 6:**
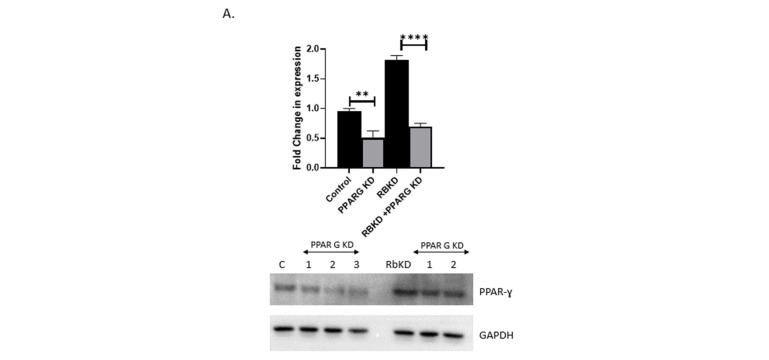

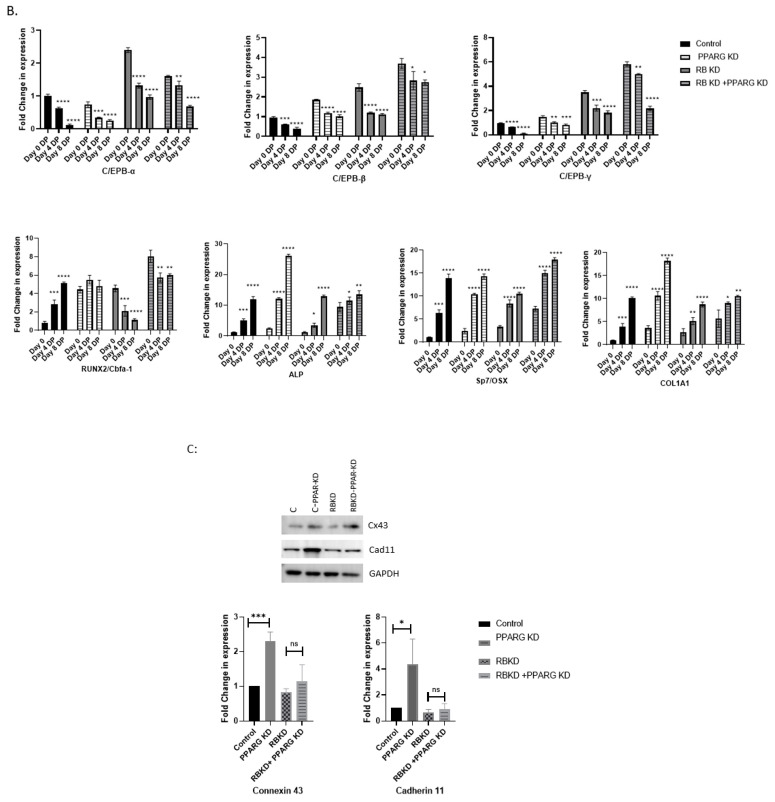

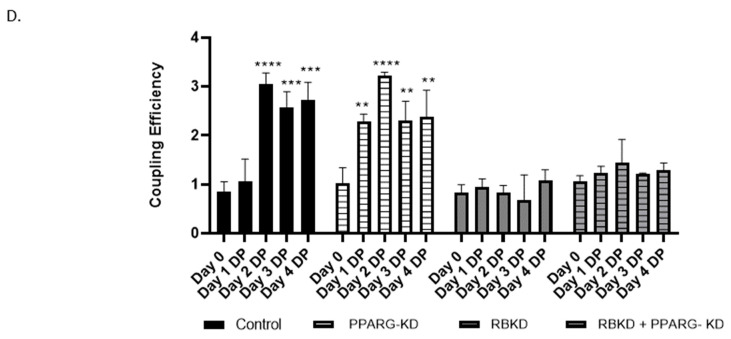
(**A**) Generation of clones containing stable knockdown of PPAR-ɣ levels. Control and RbKD cells were used to knockdown PPAR-ɣ levels. Clones with reduction in PPAR-ɣ were pooled and expanded. Analysis of these clones showed a loss of 40–50% of endogenous PPAR-ɣ expression. A representative blot and quantitation of three separate measurements are indicated. **** *p* < 0.0001, ** *p* < 0.01 compared with the respective controls. (**B**) Loss of PPAR-ɣ expression reduces adipocytic differentiation (upper panel) and increases osteoblastic differentiation markers (lower panel). Analysis using three independent clonal populations of cells with PPAR-ɣ loss in control and RbKD cells after exposure to DP media for the indicated time is shown. Data are the mean and standard deviation of three separate experiments **** *p* < 0.0001, *** *p* < 0.001, ** *p* < 0.01, * *p* < 0.05 compared with day 0. (**C**) Cx43 and Cad11 expression after PPAR-ɣ knockdown. A representative Western blot is shown, and a composite of several Western blot data quantitation is indicated below. *** *p* < 0.001, * *p* < 0.05, ns—not significant compared with day 0. (**D**) GJIC is unaffected by loss of PPAR-ɣ expression. The dye transfer assay was used to observe GJIC changes. Experiments were performed in triplicate and repeated thrice. Other clones were also tested, and similar data was obtained. **** *p* < 0.0001, *** *p* < 0.001, ** *p* < 0.01 compared with day 0.

One of the major changes that occur with the loss of Rb1 expression is the increase in adipocyte master transcription factor PPAR-ɣ. As this key change can potentially change the cell’s fate, we determined if a reduction in these protein levels might prove sufficient to reverse the changes we observed. We, therefore, attempted to stably reduce the levels of this factor in our cells. We created several clones with a stable reduction of PPAR-ɣ levels in control and RBKD cells, as described in the methods section. As shown in Figure 6A, we were able to reduce PPAR-ɣ levels to about 50% of the control levels. Cells with RBKD had higher PPAR-ɣ levels to start with. We were able to generate clones with a comparative reduction in PPAR-ɣ, albeit slightly higher than seen with the control cells. These cells were subjected to realtime PCR to determine changes to adipocytic and osteoblastic-specific gene expression. As seen in Figure 6B, the reduction in PPAR-ɣ levels was able to significantly reduce adipocyte markers tested in the control and RbKD cells (top panel). As expected, most of the osteoblast marker gene expression showed an increase with PPAR-ɣ loss (bottom panel).

When we compared changes in the expression of membrane proteins Cx43 and Cad11 after PPAR-ɣ KD using Western blot analysis (Figure 6C). We saw a small, but significant, increase in the expression of Cx43 and Cad11 in the control cells with PPAR-ɣ KD, while RbKD cells with PPAR-ɣ KD did not fare as well. Several Western blots were screened with the different clones and the composite quantitative analysis of the blots showed a significant increase in the expression of both proteins with PPAR-ɣ loss in the control cells but not in the RBKD cells (Figure 6C).

When GJIC during osteoblast differentiation was tested using the dye transfer assay, we found that there was no significant change in communication between cells with PPAR-ɣ loss in control cells (Figure 6D). The usual increase that we have seen during communication was retained in these cells. As noted earlier, the RBKD cells showed little change in communication during the differentiation process and a similar result was obtained with the double knockdowns (RBKD + PPAR-ɣ KD), where there was no significant reversal of communication to normal, as we had hoped (Figure 6D). These cells resembled RBKD in their lack of communication. These results show that while a reduction in PPAR-ɣ was sufficient to restore osteoblastic markers of gene expression, membrane proteins and communication did not show a similar reversal, suggesting that the increase in PPAR-ɣ with RbKD may not be solely responsible for these changes.

## 4. Discussion

Osteosarcomas are the most frequent primary bone cancer in children and adolescents. Many syndromes predispose individuals to osteosarcomas, including retinoblastoma syndrome due to RB1 gene mutations, Li-Fraumeni syndrome due to p53 mutations, and others resulting from various gene mutations [46]. P53 alterations are most frequently seen in 30–60% of osteosarcomas [47,48], and a similar mutation frequency is seen in the *RB1* gene [49]. RB1 gene mutations are present in 70% of all pediatric osteosarcoma patients [49]. Therefore, mutations of both genes are common in human osteosarcomas [50]. Among the functions of the RB gene, its role in the regulation of cell cycle progression is well characterized [51] and helps explain the increased incidence of osteosarcoma in adolescent patients occurring during their growth spurt. It is now clear that the role of RB1 is much vaster, ranging from cell fate, tissue differentiation, morphogenesis, senescence, self-renewal, genomic stability to apoptosis, and many others, as has been outlined in recent reviews [10,52]. To understand molecular changes in osteosarcoma, we have attempted to create a deficiency of Rb1 in osteoblasts and study how it affects normal growth and differentiation. We found that loss of Rb1 caused an increase in bone differentiation, as evidenced by increased levels of bone-specific transcription factors to much greater levels, a phenomenon that has been described in in vivo studies using acute ablations of the Rb1 gene [32]. Loss of Rb1 is also associated with an increase in adipocyte phenotype, which has been attributed to be a default pathway, as the presence of this gene is essential for the formation of osteoblasts and bone tissue [11,32].

Among the cell types in the body, the skeletal system has an extensive distribution of gap junctions in all its component cells, but particularly abundant in osteoblasts and osteocytes [53,54]. Though several types of connexins are known, Cx43 (GJA1) is most ubiquitously expressed in all types of bone cells and is involved in maintaining GJIC [20]. The importance of GJIC during osteoblast differentiation [20], and that Cx43 expression and GJIC is required for transcriptional activity of osteoblast-specific genes are also well established [55,56,57]. The most intriguing observation for us was how GJIC and the presence of Rb1 was necessary for the maintenance of the osteoblast phenotype. In both cases, a loss of their respective functions produced a shift to the adipocytic phenotype. This prompted us to determine if there was a direct role for Rb1 in mediating cell fate through control of GJIC. Our results suggest that Rb1 loss directly affects the ability of osteoblasts to maintain gap junction communication and the expression of Cx43 during osteoblast differentiation. This loss does result in a change to the cell’s behavior as it is associated with an increase in adipocytic gene expression. These results are consistent with a previous study that has utilized mouse models with targeted deletion of Rb1 to demonstrate the loss of osteoblast cell fate during mesenchymal tissue development [11] These investigators show cell lineage plasticity between adipocyte and osteoblast-specific functions to be determined by the presence and absence of Rb1. Since pRb1 is important for potentiating the activity of the osteoblast master regulator Cbfa1/Runx2 while inhibiting PPAR-ɣ [11], we hoped to reverse the changes we had observed with Rb1 loss. We found the reduction of PPAR-ɣ to be sufficient in improving the osteoblast-specific gene expression and reducing the adipocytic gene expression in control cells with endogenous PPAR-ɣ activity, and a similar reversal of phenotype was also evident in Rb1-deficient cells. However, the changes that occurred in membrane proteins, such as Cx43 and Cad11, were not reversed with a decrease in PPAR-ɣ in RbKD cells. This suggested that even though a similar mechanism of reduction in Cx43 is known to be present during adipocyte differentiation, it is not mediated by PPAR-ɣ. The presence of a normal complement of Rb1 genes in control cells also allowed for a significant increase in these proteins, which suggests the need for Rb1 in the regulation of these membrane proteins. There is sufficient evidence in the literature implicating a role for Rb1 in the regulation of membrane-specific proteins. A loss of Rb1 has been shown to lead to an expansion of calvarial osteoblasts with low expression of N-cadherin [58] and properties consistent with an immature progenitor cell capable of adipocyte osteoblast differentiation. Other adhesion-related proteins are known to be affected by Rb1 loss [12]. While these changes are seen in normal cells and tissues, tumors with RB1 loss are also known to be aggressive relative to tumors with wild-type Rb. Osteosarcomas have a higher prevalence of RB1 gene mutations and are consistently high-grade and poorly differentiated, with many showing detectable metastasis [1,59]. These are thought to result from disruptions to membrane proteins, such as cadherins and integrins constituting the epithelial-mesenchymal transition (EMT), during oncogenic progression.

While Rb’s role in regulating membrane protein integrity is well understood, it is also known that adipocyte differentiation is associated with changes to membrane, extracellular matrix and other cytoskeletal proteins occurring with cell shape alterations that accompany this process [60]. A decrease in Cx43 expression and a reduction in GJIC are also known to occur during adipocyte differentiation [42,43]. It remains to be seen if Rb1 is involved in this process. It is likely that Rb1 may mediate these effects as part of its function in maintaining differentiation and lineage plasticity in tissues [52]. However, the role of Rb1 in the expression of Cx43 and its subsequent effect on GJIC is a novel finding that has not been previously reported and requires further investigation to understand if it is a direct effect of the Rb1 pathway on cellular communication.

## 5. Conclusions

In this work, we demonstrate a reduction in gap junctional intercellular communication and Cx43 expression that accompany Rb1 gene deficiency in osteoblasts. These changes are also associated with features of dedifferentiation or trans-differentiation of osteoblasts that now display greater expression of adipocyte markers. We found a reduction of PPAR-ɣ expression to reverse the adipocyte phenotype in both control and Rb1-deficient osteoblasts, but not the changes to proteins Cx43 and Cad11. GJIC is also not reversed in Rb1deficient osteoblasts by PPAR-ɣ knockdown. These results suggest the need for further investigations to determine how Rb1 might affect gap junctional intercellular communication in osteoblasts.

## Figures and Tables

**Table 1:** Primer sequences used.

Primer	Forward	Reverse
Sp7	ACTCATCCCTATGGCTCGTG	GGTAGGGAGCTGGGTTAAGG
RUNX2	AACAAGACCCTGCCCGTG	TGAAACTCTTGCCTCGTCCG
FABP4	CCGCAGACGACAGGAAGGT	AGGGCCCCGCCATCT
Cad11	AGACGTTGGATCCGAGAAAGAG	GGTTGTCCTTCCAGGATACT
Cn43	ACAGCGGTTGAGTCAGCTTG	GAGAGATGGGGAAGGACTTGT
ALP	GCTGATCATTCCCACGTTTT	CTGGGCCTGGTAGTTGTTGT
COL1A1	ACGTCCTGGTGAAGTTGGTC	CAGGGAAGCCTCTTTCTCCT
C/EBPα	CAAGAACAGCAACGAGTACCG	GTCACTGGTCAACTCCAGCAC
C/EBPβ	GGGTTGTTGATGTTTTTGGTTT	GAAACGGAAAAGGTTCTCAAAA
C/EBPδ	CGACTTCAGCGCCTACATTGA	GAAGAGGTCGGCGAAGAGTT
PPARγ	TGAAAGAAGCGGTGAACCACTG	TGGCATCTCTGTGTCAACCATG
β-Actin	TGTCCACCTTCCAGCAGATGT	AGCTCAGTAACAGTCCGCCTAG

## Data Availability

Data is contained within the article and supplementary materials.

## Supplementary Materials

The following supporting information can be downloaded at: https://www.mdpi.com/article/10.3390/biology13010039/s1.

## References

[1] Ottaviani G., Jaffe N. (2009). The etiology of osteosarcoma. Cancer Treat. Res..

[2] Chandar N., Billig B., McMaster J., Novak J. (1992). Inactivation of p53 gene in human and murine osteosarcoma cells. Br. J. Cancer.

[3] Chandar N., Campbell P., Novak J., Smith M. (1993). Dependence of induction of osteocalcin gene expression on the presence of wild type p53 in a murine osteosarcoma cell line. Mol. Carcinog..

[4] Chen H., Hays E., Liboon J., Neely C., Kolman K., Chandar N. (2011). Osteocalcin gene expression is regulated by wild type p53. Calcif. Tissue Int..

[5] Venkatachalam S., Shi Y., Jones S.N., Vogel H., Bradley A., Pinkel D., Donehower L.A. (1998). Retention of wild type p53 in tumors from p53 heterozygous mice: Reduction of p53 dosage can promote cancer formation. EMBO J..

[6] Chandar N., Donehower L., Lanciloti N. (2000). Reduction in p53 gene dosage diminishes differentiation capacity of osteoblasts. Anticancer Res..

[7] Jacks T., Fazeli A., Schmitt E.M., Bronson R.T., Goodell M.A., Weinberg R.A. (1992). Effects of an Rb mutation in the mouse. Nature.

[8] Roufayel R., Mezher R., Storey K.B. (2021). The Role of Retinoblastoma Protein in Cell Cycle Regulation: An Updated Review. Curr. Mol. Med..

[9] Hansen M.F., Cavenee W.K. (1987). Retinoblastoma and osteosarcoma: The prototypic cancer family. Acta Paediatr. Jpn..

[10] Dyson N.J. (2016). RB1: A prototype tumor suppressor and an enigma. Genes Dev..

[11] Calo E., Quintero-Estades J.A., Danielian P.S., Nedelcu S., Berman S.D., Lees J.A. (2010). Rb regulates fate choice and lineage commitment in vivo. Nature.

[12] Sosa-García B., Gunduz V., Vázquez-Rivera V., Cress W.D., Wright G., Bian H., Hinds P.W., Santiago-Cardona P.G. (2010). A role for the retinoblastoma protein as a regulator of mouse osteoblast cell adhesion: Implications for osteogenesis and osteosarcoma formation. PLoS ONE.

[13] Laurie N., Mohan A., McEvoy J., Reed D., Zhang J., Schweers B., Ajioka I., Valentine V., Johnson D., Ellison D. (2009). Changes in retinoblastoma cell adhesion associated with optic nerve invasion. Mol. Cell. Biol..

[14] Luan Y., Yu X.-P., Xu K., Ding B., Yu J., Huang Y., Yang N., Lengyel P., Di Cesare P.E., Liu C.-J. (2007). The retinoblastoma protein is an essential mediator of osteogenesis that links the p204 protein to the Cbfa1 transcription factor thereby increasing its activity. J. Biol. Chem..

[15] Yamanouchi K., Yada E., Ishiguro N., Nishihara M. (2007). 18alpha-glycyrrhetinic acid induces phenotypic changes of skeletal muscle cells to enter adipogenesis. Cell. Physiol. Biochem..

[16] Schiller P.C., D’Ippolito G., Brambilla R., Roos B.A., Howard G.A. (2001). Inhibition of gap-junctional communication induces the trans-differentiation of osteoblasts to an adipocytic phenotype in vitro. J. Biol. Chem..

[17] Stains J.P., Watkins M.P., Grimston S.K., Hebert C., Civitelli R. (2014). Molecular mechanisms of osteoblast/osteocyte regulation by connexin43. Calcif. Tissue Int..

[18] Stains J.P., Civitelli R. (2005). Gap junctions regulate extracellular signal-regulated kinase signaling to affect gene transcription. Mol. Biol. Cell.

[19] Stains J.P., Lecanda F., Screen J., Towler D.A., Civitelli R. (2003). Gap junctional communication modulates gene transcription by altering the recruitment of Sp1 and Sp3 to connexin-response elements in osteoblast promoters. J. Biol. Chem..

[20] Civitelli R. (2008). Cell-cell communication in the osteoblast/osteocyte lineage. Arch. Biochem. Biophys..

[21] Stains J.P., Civitelli R. (2016). Connexins in the skeleton. Semin. Cell Dev. Biol..

[22] Plotkin L.I., Davis H.M., Cisterna B.A., Sáez J.C. (2017). Connexins and Pannexins in Bone and Skeletal Muscle. Curr. Osteoporos. Rep..

[23] Upham B.L., Sovadinova I., Babica P. (2016). Gap Junctional Intercellular Communication: A Functional Biomarker to Assess Adverse Effects of Toxicants and Toxins, and Health Benefits of Natural Products. J. Vis. Exp..

[24] Czyż J., Irmer U., Schulz G., Mindermann A., Hülser D.F. (2000). Gap-junctional coupling measured by flow cytometry. Exp. Cell Res..

[25] Goldberg G.S., Bechberger J.F., Naus C.C. (1995). A pre-loading method of evaluating gap junctional communication by fluorescent dye transfer. Biotechniques.

[26] Pendleton E., Chandar N. (2017). In Vitro Differentiation of Preosteoblast-Like Cells, MC3T3-E1, to Adipocytes Is Enhanced by 1,25(OH)_2_ Vitamin D_3_. Front. Endocrinol..

[27] Mukherjee P., Winter S.L., Alexandrow M.G. (2010). Cell cycle arrest by transforming growth factor beta1 near G1/S is mediated by acute abrogation of prereplication complex activation involving an Rb-MCM interaction. Mol. Cell. Biol..

[28] Zhang C., Cho K., Huang Y., Lyons J.P., Zhou X., Sinha K., McCrea P.D., De Crombrugghe B. (2008). Inhibition of Wnt signaling by the osteoblast-specific transcription factor Osterix. Proc. Natl. Acad. Sci. USA.

[29] Ducy P., Zhang R., Geoffroy V., Ridall A.L., Karsenty G. (1997). Osf2/Cbfa1: A transcriptional activator of osteoblast differentiation. Cell.

[30] Nakashima K., Zhou X., Kunkel G., Zhang Z., Deng J.M., Behringer R.R., de Crombrugghe B. (2002). The novel zinc finger-containing transcription factor osterix is required for osteoblast differentiation and bone formation. Cell.

[31] Lee J.-E., Schmidt H., Lai B., Ge K. (2019). Transcriptional and Epigenomic Regulation of Adipogenesis. Mol. Cell. Biol..

[32] Berman S.D., Yuan T.L., Miller E.S., Lee E.Y., Caron A., Lees J.A. (2008). The retinoblastoma protein tumor suppressor is important for appropriate osteoblast differentiation and bone development. Mol. Cancer Res..

[33] Ambele M.A., Dhanraj P., Giles R., Pepper M.S. (2020). Adipogenesis: A Complex Interplay of Multiple Molecular Determinants and Pathways. Int. J. Mol. Sci..

[34] Lin D.P.L., Dass C.R. (2018). Transdifferentiation of adipocytes to osteoblasts: Potential for orthopaedic treatment. J. Pharm. Pharmacol..

[35] Hashida Y., Nakahama K.-I., Shimizu K., Akiyama M., Harada K., Morita I. (2014). Communication-dependent mineralization of osteoblasts via gap junctions. Bone.

[36] Jeansonne B., Feagin F., McMinn R., Shoemaker R., Rehm W. (1979). Cell-to-cell communication of osteoblasts. J. Dent. Res..

[37] Talbot J., Brion R., Lamora A., Mullard M., Morice S., Heymann D., Verrecchia F. (2018). Connexin43 intercellular communication drives the early differentiation of human bone marrow stromal cells into osteoblasts. J. Cell. Physiol..

[38] Batra N., Kar R., Jiang J.X. (2012). Gap junctions and hemichannels in signal transmission, function and development of bone. Biochim. Biophys. Acta.

[39] Matsuo K. (2009). Crosstalk among bone cells. Curr. Opin. Nephrol. Hypertens..

[40] Gupta A., Anderson H., Buo A.M., Moorer M.C., Ren M., Stains J.P. (2016). Communication of cAMP by connexin43 gap junctions regulates osteoblast signaling and gene expression. Cell. Signal..

[41] Azarnia R., Russell T.R. (1985). Cyclic AMP effects on cell-to-cell junctional membrane permeability during adipocyte differentiation of 3T3-L1 fibroblasts. J. Cell Biol..

[42] Umezawa A., Hata J. (1992). Expression of gap-junctional protein (connexin 43 or alpha 1 gap junction) is down-regulated at the transcriptional level during adipocyte differentiation of H-1/A marrow stromal cells. Cell Struct. Funct..

[43] Yeganeh A., Stelmack G.L., Fandrich R.R., Halayko A.J., Kardami E., Zahradka P. (2012). Connexin 43 phosphorylation and degradation are required for adipogenesis. Biochim. Biophys. Acta.

[44] Sinha K.M., Yasuda H., Coombes M.M., Dent S.Y.R., de Crombrugghe B. (2010). Regulation of the osteoblast-specific transcription factor Osterix by NO66, a Jumonji family histone demethylase. EMBO J..

[45] Strauss R.E., Gourdie R.G. (2020). Cx43 and the Actin Cytoskeleton: Novel Roles and Implications for Cell-Cell Junction-Based Barrier Function Regulation. Biomolecules.

[46] Czarnecka A.M., Synoradzki K., Firlej W., Bartnik E., Sobczuk P., Fiedorowicz M., Grieb P., Rutkowski P. (2020). Molecular Biology of Osteosarcoma. Cancers.

[47] Gokgoz N., Wunder J.S., Mousses S., Eskandarian S., Bell R.S., Andrulis I.L. (2001). Comparison of p53 mutations in patients with localized osteosarcoma and metastatic osteosarcoma. Cancer.

[48] Wunder J.S., Gokgoz N., Parkes R., Bull S.B., Eskandarian S., Davis A.M., Beauchamp C.P., Conrad E.U., Grimer R.J., Healey J.H. (2005). TP53 mutations and outcome in osteosarcoma: A prospective, multicenter study. J. Clin. Oncol..

[49] Feugeas O., Guriec N., Babin-Boilletot A., Marcellin L., Simon P., Babin S., Thyss A., Hofman P., Terrier P., Kalifa C. (1996). Loss of heterozygosity of the RB gene is a poor prognostic factor in patients with osteosarcoma. J. Clin. Oncol..

[50] Toguchida J., Ishizaki K., Sasaki M.S., Ikenaga M., Sugimoto M., Kotoura Y., Yamamuro T. (1988). Chromosomal reorganization for the expression of recessive mutation of retinoblastoma susceptibility gene in the development of osteosarcoma. Cancer Res..

[51] Sellers W.R., Kaelin W.G. (1997). Role of the retinoblastoma protein in the pathogenesis of human cancer. J. Clin. Oncol..

[52] Yao Y., Gu X., Xu X., Ge S., Jia R. (2022). Novel insights into RB1 mutation. Cancer Lett..

[53] Palumbo C., Palazzini S., Marotti G. (1990). Morphological study of intercellular junctions during osteocyte differentiation. Bone.

[54] Shapiro F. (1997). Variable conformation of GAP junctions linking bone cells: A transmission electron microscopic study of linear, stacked linear, curvilinear, oval, and annular junctions. Calcif. Tissue Int..

[55] Lecanda F., Towler D.A., Ziambaras K., Cheng S.-L., Koval M., Steinberg T.H., Civitelli R. (1998). Gap junctional communication modulates gene expression in osteoblastic cells. Mol. Biol. Cell.

[56] Schiller P.C., D’ippolito G., Balkan W., Roos B., Howard G. (2001). Gap-junctional communication is required for the maturation process of osteoblastic cells in culture. Bone.

[57] Li Z., Zhou Z., Saunders M.M., Donahue H.J. (2006). Modulation of connexin43 alters expression of osteoblastic differentiation markers. Am. J. Physiol.-Cell Physiol..

[58] Gündüz V., Kong E., Bryan C.D., Hinds P.W. (2012). Loss of the retinoblastoma tumor suppressor protein in murine calvaria facilitates immortalization of osteoblast-adipocyte bipotent progenitor cells characterized by low expression of N-cadherin. Mol. Cell. Biol..

[59] Engel B.E., Cress W.D., Santiago-Cardona P.G. (2015). The Retinoblastoma Protein: A Master Tumor Suppressor Acts as a Link between Cell Cycle and Cell Adhesion. Cell Health Cytoskelet..

[60] Smas C.M., Sul H.S. (1997). Molecular mechanisms of adipocyte differentiation and inhibitory action of pref-1. Crit. Rev. Eukaryot. Gene Expr..

