# Supplementary figures and images for: Loss of Function of the Retinoblastoma Gene Affects Gap Junctional Intercellular Communication and Cell Fate in Osteoblasts
Source: Biology (Basel). 2024 Jan 11;13(1):39. doi: 10.3390/biology13010039 (PMC10813623; doi:10.3390/biology13010039)

# Figure 3

Cx43

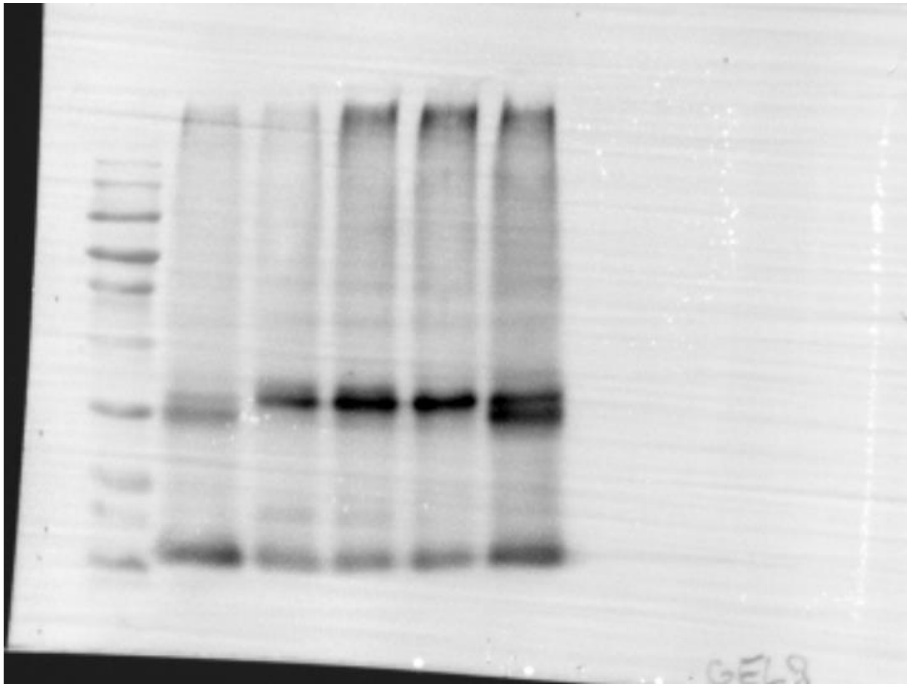

GAPDH

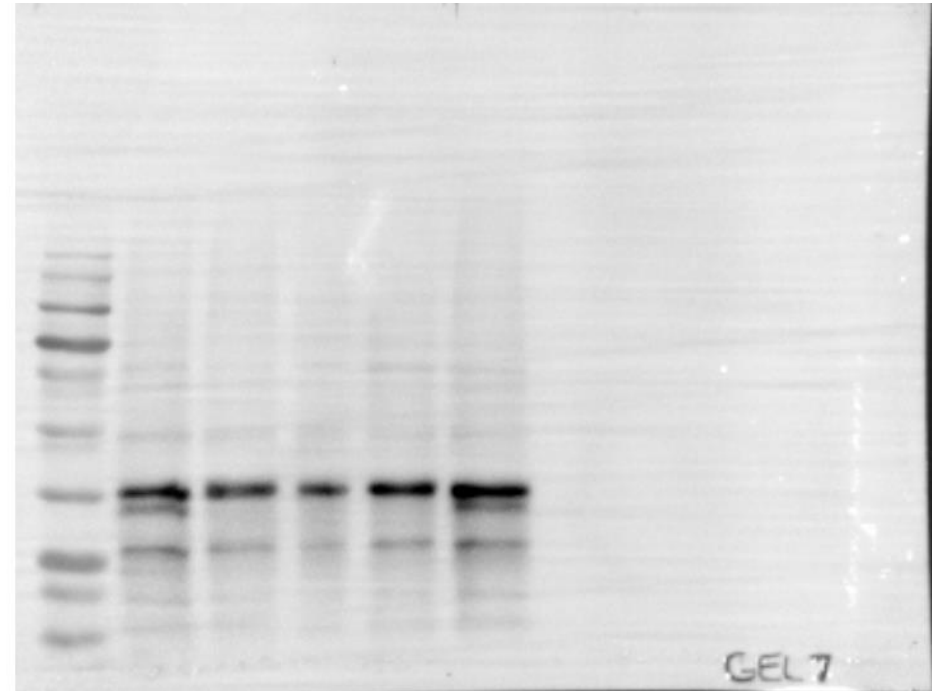

# Figure 5

Cx43

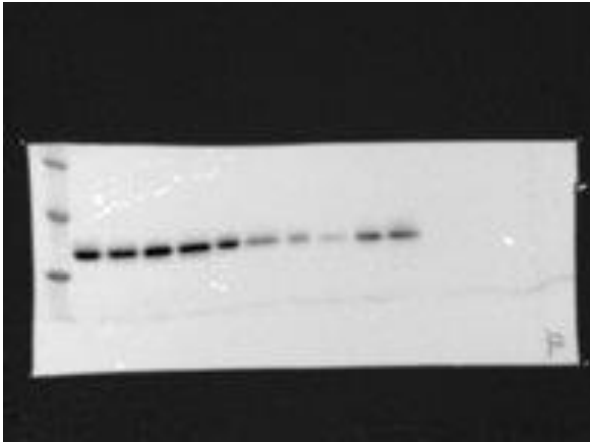

Cad11

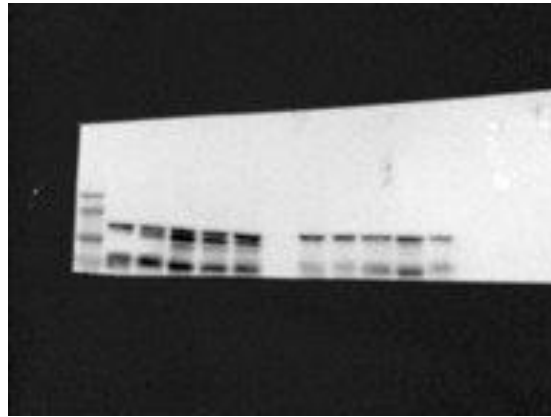

GAPDH

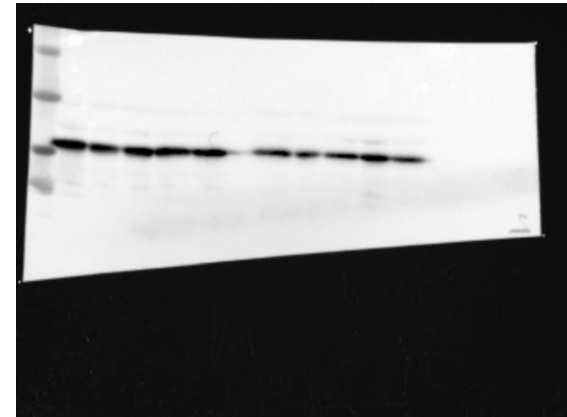

Figure 6A

PPARG

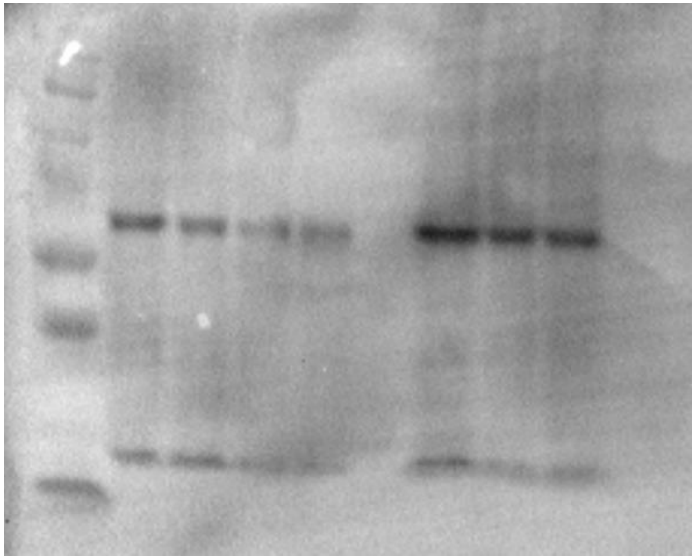

GAPDH

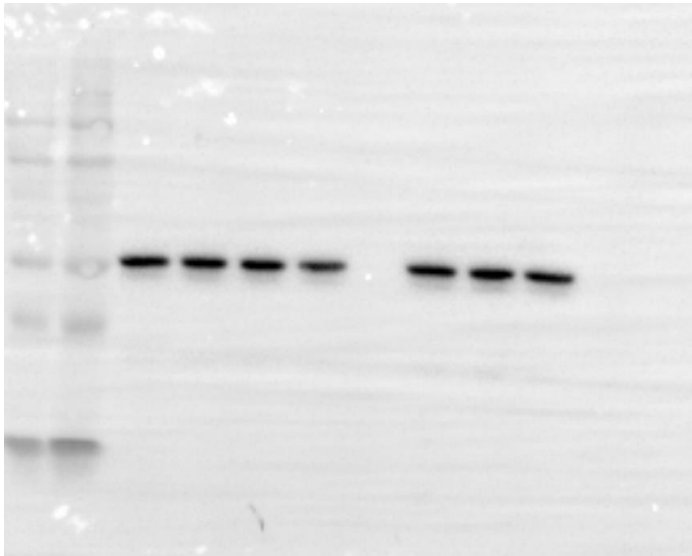

Figure 6C:

CN43

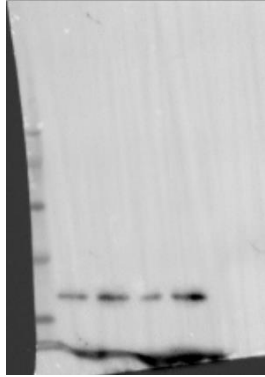

Cad11

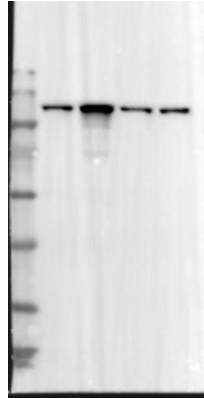

GAPDH

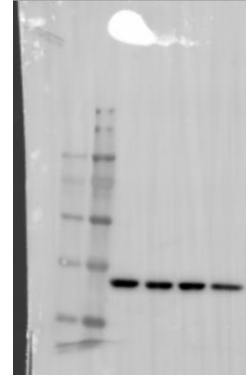

Supplement: Supplementary file 1 [file biology-13-00039-s001.zip › biology-2697594-supplementary.pdf]
